# Nutritional Quality of the Mid-Afternoon Snack of Schooled Children between the Ages of 3 and 12 Years in Three Areas in Spain

**DOI:** 10.3390/nu16121944

**Published:** 2024-06-19

**Authors:** Cristina González-Campins, Laura Ferrer Soler, Olívia Guasch-Niubó, Nadia San Onofre, Alicia Aguilar Martínez, Alba Martínez-García, Maria Manera, Gemma Salvador, Anna Bach-Faig

**Affiliations:** 1 Faculty of Health Sciences, Universitat Oberta de Catalunya, Rambla del Poblenou, 156, 08018 Barcelona, Spain; cgcampins@gmail.com (C.G.-C.); lferrerso@uoc.edu (L.F.S.); guascho@uoc.edu (O.G.-N.); 2 Department of Community Nursing, Preventive Medicine, Public Health and History of Science, University of Alicante, 03690 Sant Vicent del Raspeig, Spain; alba.martinez@ua.es; 3 FoodLab Research Group, Faculty of Health Sciences, Universitat Oberta de Catalunya, Rambla del Poblenou 156, 08018 Barcelona, Spain; aaguilarmart@uoc.edu (A.A.M.); abachf@uoc.edu (A.B.-F.); 4 Agència de Salut Pública de Catalunya, Departament de Salut, Generalitat de Catalunya, 08005 Barcelona, Spain; maria.manera_ext@gencat.cat (M.M.); gemma.salvador@gencat.cat (G.S.)

**Keywords:** snacks, dietary adequacy, feeding behavior, dietary habits, family caregiver, healthy diet

## Abstract

Background: The aim of this study was to analyze the nutritional quality of mid-afternoon snacks for schooled children aged 3 to 12 years in three areas of Catalonia (Spain). Methods: A descriptive observational study collected information on habits and the mid-afternoon snack of 782 schooled children aged 3 to 12 years in three cities, Barcelona, Girona, and Lleida, located in Catalonia (Spain). The children’s families voluntarily agreed to complete an online questionnaire that collected information about demographic data and snacking habits in the afternoon, as well as a record of mid-afternoon snack intake over three school days. Results: A total of 2163 mid-afternoon snacks were analyzed from a sample of 764 families with 3 to 12 year-old children. Sandwiches emerged as the most prevalent choice, accounting for 41.89%, followed by pastries at 23.86%, fruit at 14.38%, and a combination of fruit and pastries at 6.29%. Of the mid-afternoon snacks recorded, 22.19% were healthy, 20.90% were quite healthy, 12.85% were quite unhealthy, and 44.06% were unhealthy. Conclusions: The nutritional quality of mid-afternoon snacks for a large majority of schooled children should be improved. It is essential to develop food education programs to improve the quality of this intake from early childhood and to consider it as an opportunity to adjust the daily dietary requirements of Spanish children.

## 1. Introduction

Unhealthy dietary patterns based on ultra-processed and high-calorie foods, a low consumption of plant rich foods, and a sedentary lifestyle lead to an increase in body weight, which represents a major risk factor in the development of non-communicable diseases [1]. Currently, the prevalence of excess weight is a pressing public health concern, particularly in early life stages [2]. Recent studies highlight the association between obesity and poor dietary habits, particularly evident in different meals, especially in mid-morning snacks and breakfasts [3,4]. Several studies [3,5,6,7,8] focusing on the dietary patterns of European children and adolescents confirm a decline in diet quality in recent decades due to the increased consumption of refined foods, pastries, and fast foods along with a decrease in Mediterranean foods such as legumes, fruit, and fish. Research on the pattern of consumption at breakfast and mid-afternoon snack times confirms the unhealthy nutritional quality of the foods eaten at these periods [9,10,11,12]. The highest sugar intakes occur during these periods, with intakes reaching up to three times the daily recommended maximum of 20–25 g [13]. Additional research underscores issues with snacks, including high consumption of unhealthy and ultra-processed foods, minimal fresh foods such as fruit intake, and a significant percentage of children skipping this meal, with worsening habits tending to be observed as they age [10,14,15]. As a consequence, intakes of cholesterol, fiber, and some vitamins and minerals are also inadequate. The dietary habits of young populations in Europe, coupled with a high prevalence of other unhealthy lifestyles, contribute to the high rates of childhood obesity in Spain [16]. 

In a global context, according to the European Regional Obesity Report 2022 [16], more than half of adults and almost a third of children are overweight or obese, making it the fourth most common risk factor for non-communicable diseases. In 2016, the WHO published the Report of the Commission to End Childhood Obesity [17], highlighting the importance of healthy diets and physical activity from an early age in different global contexts. On the other hand, the Global Strategy for women’s, children’s and adolescents’ health 2016–2030 [18] also emphasized the need for adequate nutrition from childhood to maintain good health throughout the life cycle. 

In this context, recommendations underscore the importance of not restricting meals for overweight children or forcing underweight children to eat. For a balanced and nutritious diet throughout the school day, it is advisable to incorporate four or five moderate meals, including three main meals (breakfast, lunch, and dinner) and two snacks (mid-morning breakfast and mid-afternoon snack). In order to improve the nutritional quality of these intakes, it is recommended to plan all meals and prioritize plant rich foods such as fresh fruits, nuts, whole grains, vegetables, and legumes. Additionally, it is suggested to reduce the consumption of animal products, especially red and processed meats, and limit saturated fats and added sodium and sugars [9]. 

Initiatives by health promotion organizations (at the national level, such as the Spanish Agency for Food Safety and Nutrition (AESAN) [19], and at the regional level, such as the Public Health Agency of Catalonia (ASPCAT) [20], as well as at the international level [21,22,23,24]) aim to raise awareness of healthy eating at school, but challenges persist, particularly in improving the nutritional composition of mid-afternoon snacks among children. Nutrition science has primarily emphasized the overall quality of the diet, focusing on assessments of main meals and even exploring breakfast skipping; in contrast, there is a limited body of scientific literature on mid-afternoon snacking among children. Thus, the aim of this study was to analyze the nutritional quality of mid-afternoon snack in a sample of families with schooled children aged 3 to 12 years in three Spanish areas in Catalonia.

## 2. Materials and Methods

### 2.1. Study Design

A cross-sectional study was conducted to collect information on the eating habits and nutritional quality of mid-afternoon snack of families with schooled children aged 3 to 12 years from schools in three areas of Catalonia (Barcelona, Lleida, and Girona) using an online survey designed ad hoc.

In order to evaluate the survey’s validity of content, the initial proposal was discussed with a group of 12 experts made up of 80% doctors (Ph.D.); however, all of them specialized in nutrition. These experts assessed the relevance and appropriateness of the questions, as described elsewhere [25]. Then, the process of administering the survey to a small sample of known families was tested.

Participation was entirely voluntary; participants were provided with an information document about the research and an informed consent form in order to formalize their willingness to participate. Personal data were not used to identify the participants, but they were assigned an alphanumeric identification code in order to guarantee their anonymity throughout the study. 

The study was approved by the Ethics Committee of the Universitat Oberta de Catalunya (CE21PR04) to ensure compliance with the ethical principles relating to research involving human subjects and the processing of personal data set out in the UNESCO Universal Declaration of Bioethics and Human Rights [26], the Declaration of Helsinki [27], and the Belmont Report [28].

### 2.2. Study Population

The target population were parents or guardians of schooled children aged 3 to 12 years who voluntarily agreed to complete the survey on eating habits and the nutritional quality of children’s mid-afternoon snack. 

**Figure 1:**
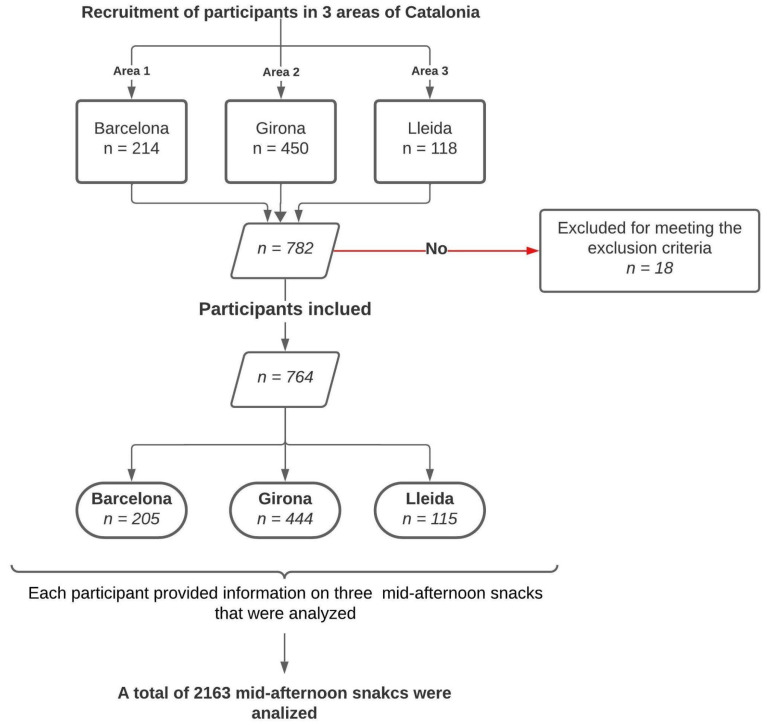
Flow diagram for recruitment of participants in the study.

The selection criteria were families with children aged 3 to 12 years, living and attending school in Barcelona, Girona, or Lleida, whose parents or guardians voluntarily agreed to the study, read and signed the informed consent form, and filled in all mandatory fields of the online form. Exclusion criteria included non-completion of the informed consent form, children outside the age range of 3 to 12 years of age, or failure to provide all the mandatory information on the form. Also excluded were those who claimed to suffer from a disease that required a special diet. Figure 1 represents the recruitment process for participants in three areas of Catalonia: Barcelona, Girona, and Lleida. Initially, we recruited 214 participants from Barcelona, 450 from Girona, and 118 from Lleida. After applying inclusion and exclusion criteria, 18 participants were excluded, leaving us with a total of 764 participants. The sample was not representative of children attending school in Barcelona, Girona, or Lleida, as the selection of participating schools and participants was conducted using the convenience sampling method.

### 2.3. Data Collection and Study Variables

This study pooled the data of three independent samples from schools in Catalonia (Spain), specifically in the cities of Barcelona, Girona, and Lleida, collected between March and April 2018 and 2022. 

The ad hoc questionnaire included an informed consent form and questions collecting information on demographics and mid-afternoon snacking habits during the school days and weekend, as well as a record of mid-afternoon snack intake over three non-consecutive school days. The form also included instructions about how the participants must include the information in the record. The variable age was categorized into three strata (3–5, 6–8, 9–12 years). 

**Figure 2:**
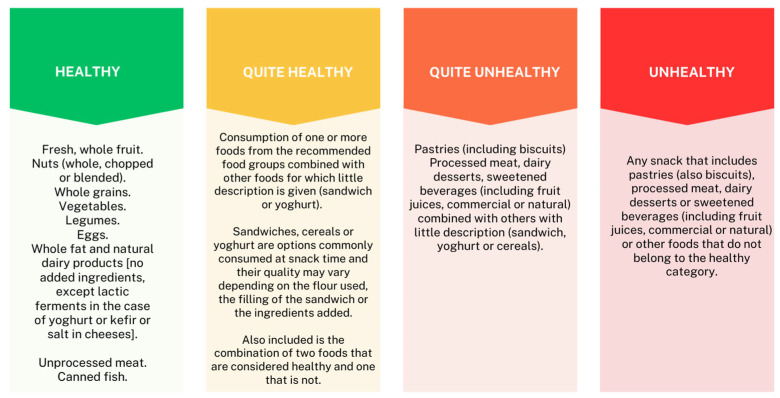
Categorization of the nutritional quality of mid-afternoon snacks.

In order to study the nutritional characteristics of mid-afternoon snacks, a review of the existing scientific evidence and official public health nutrition recommendations on mid-afternoon snacks was initially carried out. In addition, an electronic search using four major health sciences academic databases (Google Academic [Google LLC, Mountain View, California, United States], PubMed® [National Center for Biotechnology Information at the U.S. National Library of Medicine, Bethesda, Maryland, United States], Scopus [Elsevier, Amsterdam, Netherlands], Cochrane [Cochrane Collaboration, London, United Kingdom], and Web of Science [Clarivate Analytics, London, United Kingdom]) was conducted by combining different keywords (mid-afternoon snack, dietary patterns, children, childhood, toddlers). The search included literature published from 2005 to 2021, and only articles in English and Spanish were selected. Specifically, the criteria for assessing the quality of snacks were drawn from reference documents such as ASPCAT’s guidelines for healthy breakfasts and mid-afternoon snacks [20], the healthy eating guide for families of schooled children [29], and various international guides published by official organizations [21,22,23,24]. These sources provide recommendations on the composition of a healthy snack. We defined four categories (healthy, quite healthy, quite unhealthy, and unhealthy) based on the review of the existing scientific evidence on mid-afternoon snacks. This four-category classification (Figure 2) was used to analyze the nutritional quality of each mid-afternoon snack.

To analyze the nutritional quality of the mid-afternoon snacks, the study included two distinct analyses. First, the nutritional quality of all the mid-afternoon snacks was assessed. Second, the nutritional quality of the mid-afternoon snacks was evaluated on a per-participant basis. 

Initially, we analyzed the composition of each mid-afternoon snack recorded by the participants who confirmed that they had had a snack after school days. Of those that included a sandwich, the composition of the sandwich was analyzed according to the type of filling, but it was not possible to discriminate whether the bread was made with wholemeal or refined flour. The beverage choice of participants who reported consuming a drink with their snack was also documented. 

Next, based on the analysis of the composition of each mid-afternoon snack recorded by the participants, we categorized the nutritional quality of the snacks into four categories (Figure 2). To calculate the average nutritional quality of the mid-afternoon snack per participant, the values of the three mid-afternoon snacks recorded for each participant were summed and then averaged based on the categories outlined in Figure 2.

A healthy mid-afternoon snack model adapted to the habits, practices, and customs of the Spanish population was developed by reviewing available and updated dietary recommendations that included aspects of snacking. The design of the healthy mid-afternoon snack model was made with Adobe Illustrator^®^ software (version 24.3).

### 2.4. Analysis

The data were entered into a database and analyzed using SPSS^®^ version 22.0 statistical software. A descriptive analysis of the variables under study was performed. The Kolmogorov–Smirnov test was used to analyze whether the variables followed a normal distribution. Categorical variables are expressed as absolute numbers and percentages, and continuous variables as means and standard deviation. An analysis of the association of categorical variables was carried out using Pearson’s Chi-square test. In the case of the association between a categorical variable and a continuous variable, Student’s t-test was used. *p*-values of less than 0.05 were considered statistically significant.

## 3. Results

### 3.1. Nutritional Quality of Mid-Afternoon Snacks

A sample of 764 participants from Barcelona, Girona, and Lleida allowed 2163 mid-afternoon snacks to be analyzed as described in Figure 1.

In the analyzed final sample, there were 382 boys (50%) and 382 girls (50%). The mean age was 8.01 years (SD = 2.350). The age group distribution comprised 15.18% for the youngest age group (3–5 years), 40.18% for the 6–8 year group, and 44.63% for the 9–12 year group.

About 94.37% of the participants (*n* = 721) reported snacking after school (93.98% boys; 94.76% girls) and 71.99% (*n* = 550) snacked most weekends (70.68% boys; 73.30% girls). Statistical analysis showed no significant differences between the mid-afternoon snacking habits and the gender of the participants (*p* = 0.638). In relation to adherence to the habit of mid-afternoon snacking, both after school and at the weekend, differences by age were observed: 95.72% of children aged 3 to 5 years had a snack after school compared to 98% of those aged 6 to 8 years and 90.63% of children aged 9 to 12 years (*p* < 0.0001). The practice of mid-afternoon snacking over the weekends persisted in 83% of children under the age of 9, compared to 66.90% in the older age group (*p* < 0.0001).

**Figure 3:**
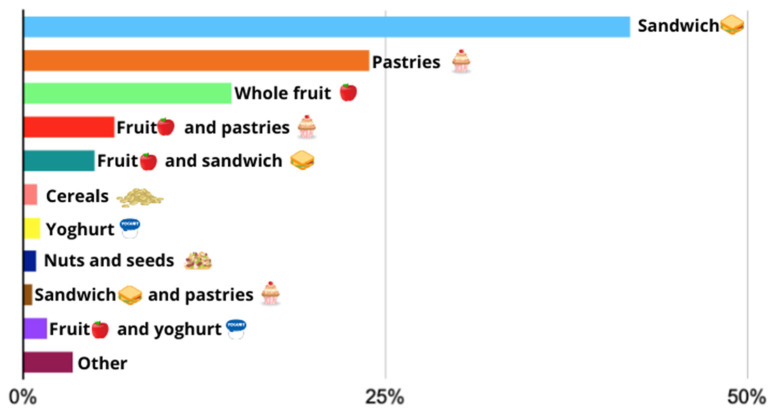
Most frequent food products or combinations in the mid-afternoon snack.

A total of 2163 mid-afternoon snacks consumed by the 721 participants over three school days were analyzed, and it was found that sandwiches were the most consumed option (41.89%), followed by pastries (23.86%), whole fruit (14.38%), and a combination of fruit and pastries (6.29%). Figure 3 shows the 11 most frequent food products or combinations of them. The analysis of the composition of the sandwiches showed that more than half (57.74%) were made with bread and processed meat, 23.53% with chocolate (solid or spreadable), and the rest with cheese (15.79%), tuna (2.17%), omelet (0.46%), or vegetable pâté (0.31%).

Colors and percentages: blue (sandwich, 41.89%), orange (pastries, 23.86%), green (whole fruit, 14.38%), red (fruit and pastries, 6.29%), dark green (fruit and sandwich, 4.95%), pink (cereals, 0.97%), yellow (yoghurt, 1.16%), navy blue (nuts and seeds, 0.88%), brown (sandwich and pastries, 0.60%), purple (fruit and yoghurt, 1.62%), garnet (other, 3.42%).

With regard to the beverage accompanying the mid-afternoon snack, 79.97% of the respondents reported drinking a liquid. A total of 611 drinks were analyzed and 51.39% of them were water, 21.28% fruit juice, 13.58% milk, the rest chocolate milkshake (9.33%), liquid yoghurt (4.09%), soda (0.16%), and a vegetable drink (0.16%).

After analyzing the 2163 mid-afternoon snacks according to the classification described in Figure 2, the results showed that 22.19% of them consisted of healthy options, 20.90% were quite healthy, 12.85% were quite unhealthy, and 44.06% were unhealthy. The average of the mid-afternoon snack nutritional quality of each participant’s was also assessed according to the aforementioned classification (Table 1) and averaged. It was found that almost half of the participants’ snacks (42.90%) were categorized as unhealthy, whereas for the rest of the participants, they were categorized as quite unhealthy (25.20%), quite healthy (21.80%), and healthy (10.11%). The analysis of the average mid-afternoon snack nutritional quality by gender (Table 1) showed no statistically significant differences (*p* = 0.968). In relation to age, the unhealthy mid-afternoon snack was the most frequent in all three age groups, with a positive correlation as the age of the child increased (from 30.6% in the 3–5 year age group to 53.10% in the 9–12 year age group (*p* < 0.0001)) (Table 1).

### 3.2. Healthy Mid-Afternoon Snack Model

Table 2 shows a summary of the organizations or official bodies that include recommendations on mid-afternoon snacks and breakfasts in their dietary guidelines or other documents aimed at the general population. It highlights the importance of prioritizing plant-based foods such as fresh fruit, nuts, whole grains, vegetables, and legumes, and of avoiding products with high amounts of salt, added sugars, or saturated fats [14,15,20,21,22,23,24,30,31,32].

**Figure 4:**
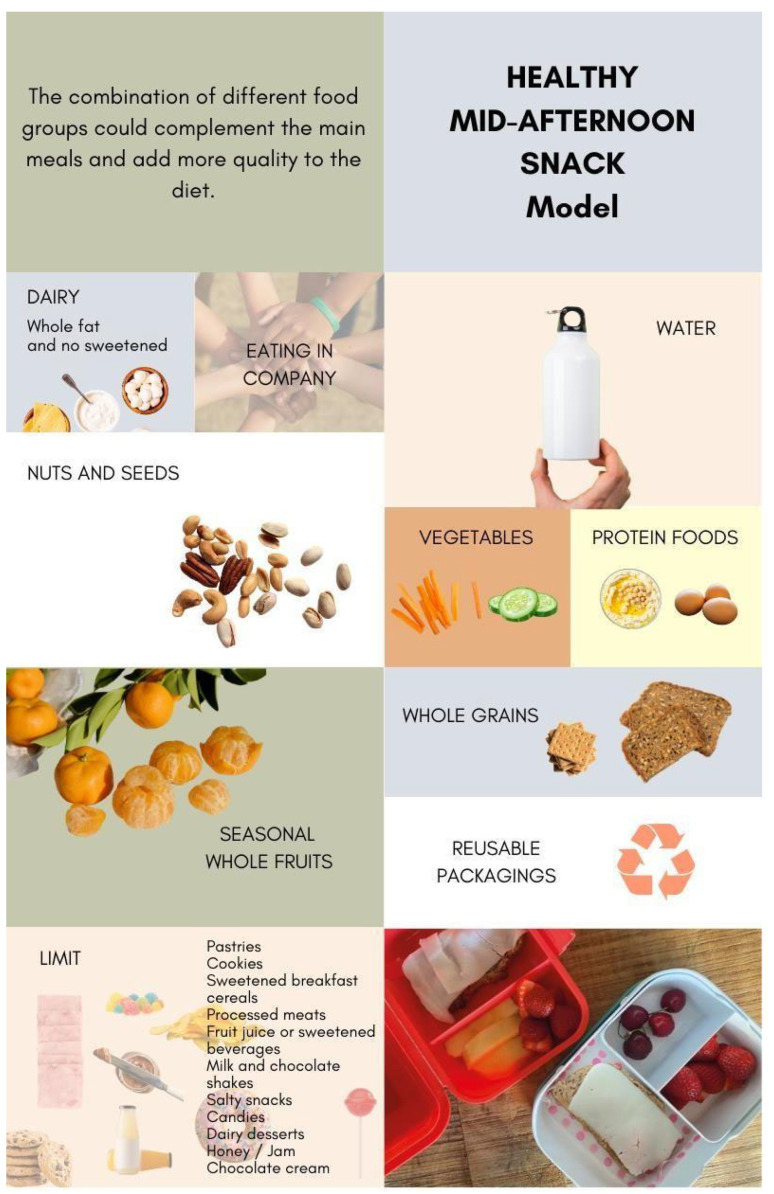
Healthy mid-afternoon snack model.

Taking into account these various recommendations, we developed the healthy snack model presented in Figure 4. The devised healthy mid-afternoon snack model (Figure 4) was divided into 13 pieces of different colors and sizes to represent the recommendations of the guidelines and documents consulted, as well as the importance of combining different food groups to configure a mid-afternoon snack, and at the same time it underscores the multifactorial nature of contemporary dietary behaviors, encompassing aspects of health, sustainability, social considerations, and satisfaction.

Seasonality: Opt for seasonal products to enhance economic viability, promote sustainability, and introduce dietary variability.

Origin: Prioritize locally sourced foods, aiming for proximity to one’s residence (within a maximum of 100 km) to reduce transportation-related environmental impacts.

Food Packaging: Refrain from selecting shakes and juices packaged in tetra bricks often, and generally limit the consumption of juices, which should not constitute a daily beverage choice. Additionally, avoid individually packaged nuts or cereals to minimize the use of single-use packaging and favor reusable containers. Adopting bulk purchasing practices represents a sustainable alternative, allowing for the acquisition of precise quantities without adherence to standardized formats (e.g., kilograms or liters of products).

Examples: Some potential healthy breakfasts and snacks in the Spanish cultural context include (a) seasonal fruit salad with nuts and wholegrain toast with olive oil and rosemary; (b) a wholegrain sandwich with vegetable stew, pine nuts, and bonito; (c) regular milk or plant-based fortified milk (respecting the family decision) and wholegrain toast with vegetable pâté or hummus; (d) natural yogurt with oats and seasonal fruit.

## 4. Discussion

The study’s findings show that 68.10% of children consume mid-afternoon snacks that are classified as either quite unhealthy or unhealthy. This indicates a significant chance for improvement by highlighting a pattern of poor food choices that is common in this population. Notably, while the overall number of snacks shows a higher percentage of healthy options, the average quality of each participant’s snack is lower because healthier options are not as common. The study is consistent with previous research [10,11,12] linking mid-afternoon snacking in children to a high consumption of ultra-processed products, refined grains [33], and a low intake of fresh foods. Consistent with prior research [31,32,33,34,35], mid-afternoon snacks are identified as nutritionally compromising, often high in energy density, salt, added sugar, and saturated fats, with variations based on factors like hunger sensation, time and place of consumption, culture, and activity. Notably, snacks consumed at home or work tend to be healthier than those eaten on-the-go, potentially explaining the lower nutritional quality of mid-afternoon snacks consumed during travel or leisure activities away from home [34,36].

Regarding the snacking habit, previous research [10,11,12,13,32] has indicated that it tends to decrease with age, a trend that aligns with the observations made in the current study. However, although no significant differences have been found, as observed elsewhere [10], it is relevant to consider gender, as girls have shown a slightly higher frequency of snacking compared to boys. Furthermore, the results emphasized the importance of paying attention to the day of the week, as snacking is more common on weekdays and decreases on weekends. In this context, previous evidence showed differences in nutrient intakes and temporal aspects of eating behavior, with extended durations observed during weekends [37,38]. Furthermore, evidence seems to corroborate that snacking in the morning versus later in the day has a greater nutritional quality.

Taking into account previous studies and the results of the present research, it could be argued that the primary challenge lies in improving the nutritional quality of mid-afternoon snacks rather than solely advocating for the adoption of snacking habits. In a larger sample of 2851 children, 78–84% typically consumed mid-afternoon snacks [10]. While 94% of our population had an after-school snack, most were unhealthy. Early childhood emerges as the optimal stage for health promotion, given its lasting impact [39,40,41]. Our results indicate age as a factor in declining snacking nutritional quality, aligning with studies noting the adolescence-related shift away from family influences on eating habits [42]. Restrictive feeding practices and unhealthy home access have an impact on children’s snacking; therefore, it is important to emphasize positive parenting practices and family meal preparation [43,44]. When children participate in meal preparation and are involved in culinary activities, it can facilitate the acceptance of new ingredients and healthy foods and establish positive role modeling [43]. Evidence underscores the influential role of family members and individuals in proximity to children [34]. Consequently, the adoption of healthy dietary practices by these influential figures facilitates the natural transmission of such habits [43]. Encouraging seasonal, fresh snacks can instill a lifelong preference for healthy foods [45]. Promoting healthy eating, especially through suitable snacks, should target families, encompassing parents, grandparents, and caregivers. Studies show that increased consumption of healthy snacks is correlated with parental habits, family nutrition knowledge, and the availability of healthy foods [46]. Conversely, practices like unhealthy home options and permissiveness relate to unhealthy food intake, associated with high body weight [45]. Positive feeding practices, especially in socio-economically disadvantaged and culturally diverse households, include a regular mealtime schedule, guided food choices, modest portions, and respecting the child’s hunger and satiety cues [47].

Regarding prevalent mid-afternoon snacks, our investigation identified sandwiches as the top choice, followed by pastries, aligning with similar studies in the Spanish child population [10,11,12,48]. The analysis of the nutritional quality of these mid-afternoon sandwiches showed that their composition was mostly unhealthy. This categorization was determined by the preference for processed meat or chocolate cream, while healthier options, as recommended by official bodies [14,15,20,21,22,23,29,49,50], such as cottage cheese, canned fish, eggs or plant origin fillings, were infrequent in our sample. In the study conducted by Echevarría et al. [12], beyond examining sandwich fillings, an investigation was undertaken into the specific type of bread utilized, with industrial sliced bread and white bread emerging as the most frequently consumed varieties. The present study did not include information about the type of bread, which would have been interesting given the benefits of wholegrain cereal consumption [37,38] and the recommendations made by health promotion bodies in many countries [14,15,20,21,22,23,24,29,31,49,50]. Switching from refined cereals to whole grains is one of the aspects proposed in ASPCAT’s “Small changes for better eating” guide [50], alongside others such as selecting seasonal and locally sourced foods, prioritizing water as the primary beverage, and incorporating virgin olive oil. These changes, together with increasing the consumption of foods from plants and reducing the consumption of products with added sugars or salty, ultra-processed, red meat products, represent aspects that can significantly improve the nutrition quality and sustainability of food [50]. There is no clear answer to the science of mid-afternoon snacking in terms of its health impacts, particularly in children [51]. Depending on the composition, frequency, and timing of snacking, it could have a positive [52] or negative [33] impact on health. However, if energy and nutrient intake are distributed on 4–5 daily occasions instead of only 3 meals, the inclusion of 1–2 snacks eases possible metabolic or digestive overload as a result of fewer larger meals together with granting the ability to meet food group and nutrient recommendations such as fruits, dairy, vitamins, and fiber [52,53,54,55,56]. The inconsistency and the weak evidence could probably be attributable to the lack of a standardized definition [32,52]. Snacks contribute from 10% to 36% of daily calories, with a frequency of consumption of about two snacks per day [36,52]. According to the latest American Dietary Guidelines, snacking is considered an opportunity to supplement and adjust daily dietary requirements [22]. This recommendation is particularly interesting during childhood, when main meal portions are sometimes insufficient and the habit of eating between main meals is common [1,2,5,32,50]. The New Zealand Ministry of Health takes a similar view, considering snacking as a valuable contribution of energy and nutrients [24]. Thus, one approach to adjusting energy and meeting daily dietary requirements through snacking is to combine different food groups [20,21,22,23,29]. Moreover, there is also uncertainty between the quality of snacks and body composition in other studies [53]. Nevertheless, more research, ideally utilizing a prospective design, is essential before firm conclusions can be drawn. The limited existing studies on meal and snack eating behaviors, especially in relation to overall diet and measures of body fatness [7,9,13,55,56], have generated conflicting results, necessitating further investigation [53].

Most of the official recommendations on snacks consulted include the food groups that should be consumed and those that should be avoided or limited. All encourage the consumption of foods of plant origin and dairy products, but there are differences when it comes to specifying what foods should be included in the food groups. Depending on the geographical region and consumption habits, there is a lesser or greater preference for fruit or vegetables. With regard to nuts and wholegrain cereals, both groups are recommended in most instances. There are discrepancies when it comes to dairy products. In the United States of America, Chile, or the United Kingdom [15,22,31], choosing low-fat versions is recommended, whereas ASPCAT recommends [20,29] whole, natural, fermented, and unsweetened dairy products. In relation to the consumption of dairy products, it is interesting to note that they contain vitamins and minerals with adequate bioavailability that contribute to meeting nutritional requirements in infancy [57,58]. A recent study questions the recommendation to consume low-fat dairy products, as there are no conclusive results that the consumption of whole dairy products is related to weight gain and poor metabolic health, but there is evidence that their whole, no-added sugar, and fermented versions are favorable to health status [59]. Additional factors unrelated to the nutritional quality of snacks are considered, as they contribute to the establishment of healthy eating habits. In formulating the healthy mid-afternoon snack (Figure 4), all recommendations derived from consulted guidelines were systematically considered, with preference given to those aligning with the social, cultural, and environmental aspects. This graphic representation highlights the importance of combining different food groups, prioritizing plant-based ones, water as the drink of choice, bread and derivatives made with 100% wholemeal flour, and quality protein foods such as eggs, legumes, nuts, and seeds. It also warns about products that should be limited or avoided. Reference is also made to sustainability, in addition to emphasizing the inclusion of foods of plant origin through the use of reusable packaging and the consumption of fresh, seasonal, and local food, as well as eating in company and in an emotionally satisfying way. Not to be forgotten is the prioritization of minimally processed foods, those with a lower NOVA level [60], and foods beneficial for planetary health [61,62]. This model is intended to be a useful tool for future interventions to promote the consumption of healthy mid-afternoon snacks among the Spanish child population.

In the Spanish context, in order to make these dietary intakes more healthful and sustainable, it is recommended to strategize all meals by prioritizing fresh fruits, nuts, whole grains, vegetables, and legumes [29]. The prevalence of foods rich in plant sources is a distinctive trait of the Mediterranean diet, necessitating a concurrent reduction in the consumption of animal products, particularly red and processed meats [30]. Research on the dietary patterns of preschool-aged children indicates that a considerable proportion fail to meet the recommended intake levels of fruits and vegetables [8,63]. 

**Figure 5:**
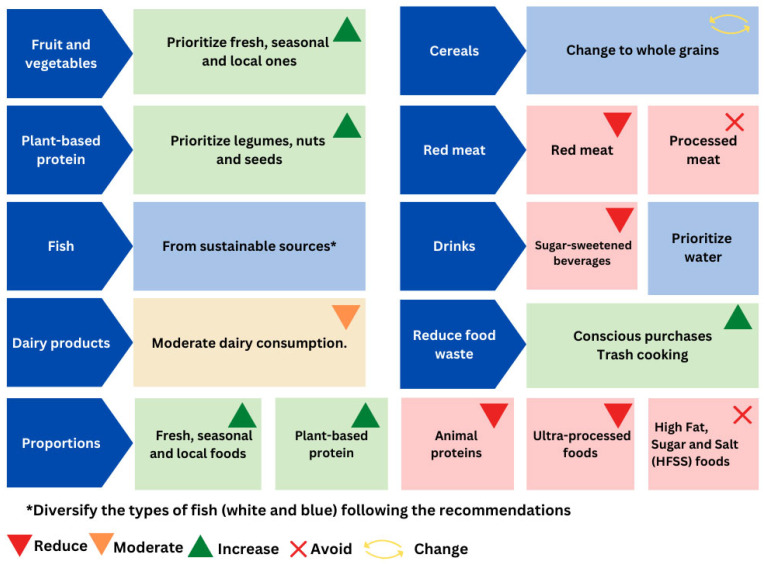
Recommendations for Healthy and Sustainable Foods (adapted from The Association of UK Dietitians One Blue Dot, 2020, and the Public Health Agency of Catalonia (ASPCAT), 2019).

In the Spanish context, the Mediterranean diet facilitates ensuring the adequate intake of nutrients and preventing non-communicable chronic diseases and also exhibits a low environmental footprint [30]. In addition, in terms of the foods that should be prioritized, Figure 5 was conceived to summarize the recommendations for healthy and sustainable foods [50,64]. Because, although healthy eating was the main focus in the school stage until a few years ago, it is now necessary to add sustainability criteria and align with the recent Food Based Dietary Guidelines in Spain that incorporate the food sustainability dimension [65]. This implies that, instead of solely focusing on what we eat, attention is also paid to how we eat.

On the other hand, it is essential to have institutional support for the development of food education programs that raise awareness of the importance of mid-afternoon snack quality and place value on snacking as part of childhood eating. Promoting healthy habits during childhood necessitates a collaborative effort, and it is pertinent to incorporate additional stakeholders who play integral roles in shaping children’s dietary choices, including schools, public health agencies, and other community entities. Schools serve as crucial educational platforms, incorporating health education into the curriculum and fostering environments that align with healthy snack guidelines. Public health agencies bring expertise in policy development, collaborating with schools to establish evidence-based guidelines that support national health objectives [14,15,20,21,22,23,24,29,31,50]. This partnership extends beyond formal guidelines to encompass the cafeteria and vending machine offerings and community engagement. The collective involvement of local governments, parents, and community organizations creates a supportive ecosystem, reinforcing healthy habits both within and outside the school environment.

This study has some strengths and limitations. Firstly, there is a constraint related to its cross-sectional nature and the fact that the dietary assessment was self-reported. Secondly, with regard to the sample, although the participants in the study and number of snacks analyzed were relatively large, it should be noted that it was not representative of the children attending school in the cities of Barcelona, Girona, and Lleida, as the selection of participating schools was made using the convenience sampling method. In addition, among the participants, as has been identified in many studies, there is a bias in participation; as described elsewhere, the samples are mostly female, highly educated caregivers [43] with children’s healthy snacking behaviors. Thirdly, the restrictions imposed by the COVID-19 pandemic period had an impact on the sampling process and proximity to the participating families by not allowing physical access to schools. Another is the open-respond survey, which yielded incomplete information that did not allow us to discriminate whether the bread or derivatives were made with wholemeal or refined flour, whether the dairy products were natural or sweetened, or whether the homemade pastries had been prepared with healthy ingredients such as extra virgin olive oil, wholemeal flour, or fruit and vegetables as sweeteners. The quality of the sandwich can vary depending on the flour used, the filling, and the ingredients added to the bread, but since it was impossible to discriminate, its composition was only examined in relation to the type of filling. Although impeding a precise assessment of their nutritional quality, the open-response survey modality did, however, have some advantages since it allowed us to learn about more options or combinations of mid-afternoon snacks consumed. Moreover, examining the extracurricular activities undertaken by children after school could offer valuable insights into the alignment between the chosen snacks and the physical demands of these activities. Additionally, contextualizing snacks by investigating the timing and location of consumption (e.g., at home, while commuting, or at school) would provide a more comprehensive understanding. These considerations, encompassing activity-related and environmental factors, could enrich the depth and applicability of future research in this field.

## 5. Conclusions

The role of mid-afternoon snacks within the daily diet should not be underestimated; however, they are normally considered smaller, less formal eating occasions, although the categorization may vary based on cultural norms and individual habits.

The present cross-sectional study conducted in three areas of Catalonia, Spain, demonstrates the lower quality of mid-afternoon snacks, aligning with findings from similar studies primarily conducted in other countries [53,66]. The results of the study show that even though most kids snack on both school and non-school days, the nutritional quality of these snacks is suboptimal. The prevalent mid-afternoon snack choices commonly lack the recommended food groups outlined by official organizations. Thus, the current challenge is to improve their nutritional quality by raising awareness and facilitating all those involved in the acquisition of healthy habits in childhood through resources and access to healthy, sustainable, and satisfactory snacks to be prepared. As a practical implication, a healthy mid-afternoon snack model is proposed along with a summary of guidelines healthy and sustainable foods, which aims to become an effective resource for improving the nutritional quality of mid-afternoon snacks in the Spanish child population.

Additional research, especially with a prospective design, is required to study the nutritional quality and effect of different combinations of foods in mid-afternoon snacks on overall diet quality and health outcomes, as well as food parenting or family food environments and their relation to snacking.

## Figures and Tables

**Table 1:** Nutritional quality of mid-afternoon snacks by gender and age group.

		Nutritional Quality of Mid-Afternoon Snacks				Total n (%)	*p* Value
		U	QU	QH	H		
		*n* (%)	*n* (%)	*n* (%)	*n* (%)		
Gender	Boy	151 (42.10)	93 (25.90)	78 (21.70)	37 (10.30)	359 (100.00)	0.968
	Girl	158 (43.60)	89 (24.60)	79 (21.80)	36 (9.90)	362 (100.00)	
Age groups	3–5 years	34 (30.60)	31 (27.90)	37 (33.33)	9 (8.10)	111 (100.00)	<0.001
	6–8 years	111 (36.90)	88 (29.20)	72 (23.90)	30 (10.00)	301 (100.00)	
	9–12 years	164 (53.10)	63 (20.40)	48 (15.50)	34 (11.00)	309 (100.00)	
	Total	309 (42.90)	182 (25.20)	157 (21.80)	73 (10.1)	721 (100.00)	

U: Unhealthy. QU: Quite unhealthy. QH: Quite healthy. H: Healthy.

**Table 2:** Dietary guidelines and/or publications from official agencies that include dietary recommendations on snacks for the general population.

Country	Year *	Agency	Concepts	Recommended Food Groups	Format
Canada [21]	2021	Government of Canada	Children: opportunity to adjust daily dietary requirements. Planning. Eat consciously. Avoid products with salt, free sugars, and saturated fats.	Plant-based and low-fat dairy products.	Video
USA [22]	2020	USDA **	Children: opportunity to adjust daily dietary requirements. Correct development. Planning. Combine food groups.	Plant-based and low-fat dairy products.	My Plate (Graphic)
Europe [30]	2020	Updated Mediterranean Diet Pyramid	Sustainability. Half portions. Local production.	Nuts and seeds.	Pyramid (Graphic)
International [14]	2020	WHO	Avoid products with salt, free sugars, and saturated fats.	Plant-based and few processed foods.	Text
United Kingdom [31]	2022	British Nutrition Foundation	Half portions. Planning. Eat consciously. Avoid products with salt, free sugars, and saturated fats.	Plant-based and low-fat dairy products. Nuts and seeds. Legumes. Wholegrain cereals.	Eatwell guide (Graphic)
Catalonia [20]	2018	ASPCAT ***	Sustainability. Half portions. Combine food groups. Avoid products with salt, free sugars, and saturated fats. Conviviality. Drinking.	Plant-based food. Fermented dairy products and milk. Nuts and seeds. Wholegrain cereals. Water.	Text
Chile [15]	2016	Health Department. Government of Chile.	Half portions. Avoid products with salt, free sugars, and saturated fats. Drinking.	Plant-based food. Low-fat dairy products. Nuts and seeds. Water.	Text

* Publication or last update year. ** United States Department of Agriculture. *** Public Health Agency of Catalonia.

## Data Availability

The datasets used and/or analyzed during the current study are available from the corresponding author on reasonable request.
